# Effect of Muscle Loss but Not Fat Loss during Primary Debulking Surgery and Chemotherapy on Prognosis of Patients with Ovarian Cancer

**DOI:** 10.3390/jcm11113184

**Published:** 2022-06-02

**Authors:** Naomi Nakayama, Kentaro Nakayama, Tomoka Ishibashi, Satoru Katayama, Satoru Kyo

**Affiliations:** 1 Department of General Medicine and Community Health Science, Hyogo Medical University, Sasayama Medical Center, 5 Kurooka, Tanbasasayama 669-2321, Japan; s-katayama@hyo-med.ac.jp; 2 Department of Obstetrics and Gynecology, Shimane University, 89-1 Enya, Izumo 693-8501, Japan; tomoka19850314@gmail.com (T.I.); satoruky@med.shimane-u.ac.jp (S.K.)

**Keywords:** ovarian cancer, skeletal muscle loss, fat loss, sarcopenia, cachexia

## Abstract

Although the negative effect of muscle loss during invasive treatment has been widely reported in patients with cancer, its value in patients with ovarian cancer is not clear. Therefore, this study was conducted to clarify whether muscle loss during cytoreductive surgery and chemotherapy affects prognosis in patients with ovarian cancer. We retrospectively recruited 58 patients with ovarian cancer who underwent site reductive surgery and chemotherapy at Shimane University Hospital from March 2006 to November 2013 and for whom pre- and postoperative computed tomography were available. Skeletal muscle changes and fat mass volume during primary debulking surgery and chemotherapy were subsequently investigated at the level of the third lumbar vertebra. Muscle and fat mass loss occurred independently in half of the patients. Muscle loss, but not fat loss, was associated with disease-free survival (*p* = 0.041 and *p* = 0.794, respectively) and poor overall survival (*p* = 0.033 and *p* = 0.61, respectively). Cancer therapy is invasive and causes compositional changes in the body, such as muscle and fat loss. During cancer therapy, muscle loss, but not fat loss, may be associated with worse prognosis in ovarian cancer.

## 1. Introduction

Globally, ovarian cancer shows the poorest prognosis among gynecologic cancers [1]; its incidence has been sharply rising. Due to its silent progression and a lack of effective screening modalities, most patients have peritoneal dissemination and distant metastases by the time of diagnosis. Although combination chemotherapy with platinum–taxane resulted in an improvement in prognosis, the 5-year overall survival (OS) rate remains approximately 45%. The residual tumor and clinical stage are the factors that determine the prognosis of patients with ovarian cancer after debulking surgery and chemotherapy. Unfortunately, these are the factors that are not modified unless early detection tools and novel effective chemo-drugs are introduced [2]. Prognostic factors that have the potential to be modified by supportive intervention are expected to lead to improving patient prognosis. Sarcopenia is a disease defined by distinguished loss of skeletal muscle mass and quality. The coexistence of sarcopenia is associated with poor outcomes in various diseases, including cancer [3,4,5,6,7,8,9]. Factors related to sarcopenia are associated with the prognosis of cancers of the digestive organs, such as gastric cancer, hepatocellular carcinoma (HCC), biliary duct cancer and pancreatic cancer [10,11,12,13]. Sarcopenia can be modified by adequate nutritional intervention and physical exercise. Therefore, integrated intervention with nutritional and rehabilitative support for sarcopenia has been recommended as a supportive cancer treatment to improve patient prognosis. We previously reported that preoperative sarcopenia, such as reduced muscle mass and quality, has no significant effect on the prognosis of patients with ovarian cancer [14]. Patients with cancer undergo invasive treatments such as surgery and chemotherapy. These treatments affect body composition [15]. Several studies have reported the influence of muscle and fat mass changes during cancer therapy on the prognosis of patients with cancer [16,17]. However, there is no consistent view of the relationship between skeletal muscle and fat mass loss and prognosis in most cancers, including ovarian cancer [16,17,18,19]. In this regard, the study aimed to evaluate the changes in body composition from primary site reductive surgery through chemotherapy and further aimed to investigate the effect of changes on the prognosis of patients with ovarian cancer in the Japanese population.

## 2. Materials and Methods

### 2.1. Patients

This retrospective study evaluated 58 patients, regardless of the FIGO stage, who underwent primary cytoreductive surgery followed by chemotherapy for ovarian cancer at the Department of Gynecology at Shimane University Hospital from March 2006 to 2013. Standard surgery included total hysterectomy, bilateral salpingo-oophorectomy and omentectomy, with or without pelvic and para-aortic lymph node dissection, followed by taxane–platinum-based standardized chemotherapy. The patients underwent computed tomography (CT) imaging for clinical evaluation before surgery and after chemotherapy. Initial CT was performed within a few weeks before surgery, and a second CT was taken after primary surgery and chemotherapy within 1 year of the initial CT at our institution. 

Approval for this study protocol was obtained from the institutional ethics and research review boards of Shimane University (IRB No. 20191120-1). The study was conducted in accordance with the 1996 Declaration of Helsinki. Written informed consent was obtained from all patients in this study. 

### 2.2. Image Analysis

Plain CT slice images at the L3 level were used for the evaluation of skeletal muscle and fat mass. Skeletal muscles evaluated included the psoas, paraspinal and abdominal wall muscles. The total fat area consisted of visceral and subcutaneous fat mass. Skeletal muscle and fat areas were examined by two experienced radiologists using Horos v4.0 software (Horos group, Annapolis, MD, USA) based on tissue-specific radiodensity. Skeletal muscle, visceral fat mass and subcutaneous fat mass were distinguished by Hounsfield Unit (HU) thresholds from −29 to 150, from −150 to −50 and from −190 to −30 HU, respectively. 

### 2.3. Analyzed Parameters

Changes in muscle and fat mass between CT scans were calculated and expressed by percentages. These percentages were divided by the number of days between scans and multiplied by 100 to express percent change per 100 days as a standard measure. A measurement error was defined as 2% based on the previously reported accuracy of CT for muscle and fat mass analyses [20]. The changes were divided into a loss group (>2% decrease per 100 days) and a gain group (any increase or ≤2% decrease). 

Patients were categorized into two groups depend on the status of muscle volume and fat volume change during cancer treatment: the gain and loss groups. Then, patients’ characteristics were compared based on the following variables at diagnosis: age, body mass index (BMI), performance status, International Federation of Gynecology and Obstetrics (FIGO) stage, preoperative body composition and days of hospitalization period. Disease-free survival (DFS) and OS were analyzed based on changes in muscle and fat volume. 

### 2.4. Statistical Analyses

All data were statistically analyzed by using the SPSS 27.0 (IBM Corporation, Armonk, NY, USA) software. *p* values of <0.05 were considered as statistically significant. Differences between groups were evaluated using Student’s *t* and χ^2^ tests for continuous and categorical variables, respectively. DFS and OS in the two groups were compared using Kaplan–Meier curves and log-rank tests. Univariate analyses were performed to identify factors significantly associated with patient survival, and hazard ratios and 95% confidence intervals were calculated. 

## 3. Results

**Figure 1:**
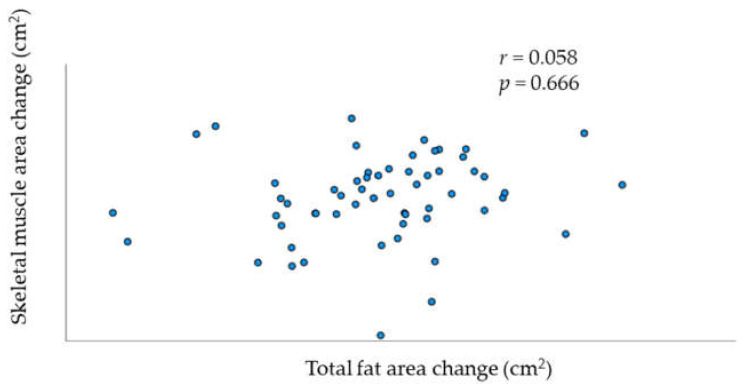
Relationship between change per 100 days in skeletal muscle and total fat.

Patient characteristics are shown in Table 1. Among the 58 patients included, 23 had the advanced-stage disease (FIGO stages III and IV). All histological types were included, and serous was the most dominant histological subtype (39.7%). The mean BMI at diagnosis was 22.73 ± 3.82 kg/m^2^. The changes in median muscle mass and fat mass after debulking surgery and chemotherapy are shown in Table 2. Treatment with primary debulking surgery and chemotherapy significantly decreased median skeletal muscle mass. The median decrease in skeletal muscle during this period was 2.55% per 100 d. Conversely, the decrease in fat mass, including total, cutaneous and visceral fat mass, was not significant. The prevalence of loss or gain in muscle or fat volume is summarized in Table 3. Table 4 shows the prevalence of patients with loss/gain of muscle/fat volume in each FIGO stage. There was no association between changes in skeletal muscle mass and total fat mass (Figure 1; r = 0.058, *p* = 0.666).

Clinicodemographic factors were classified according to the status of muscle and fat volume changes during primary debulking surgery and chemotherapy (Table 5 and Table 6). Change in muscle mass was associated with the presence of residual tumor in univariate analysis. There was no association between the status of muscle mass change with age, BMI, length of hospital stay, FIGO stage IV, performance status or body composition in the preoperative period. Patients who gained muscle mass tended to have a higher skeletal muscle index (SMI) than those who lost muscle mass. The fat mass change was not associated with age, BMI, length of hospital stay, FIGO stage IV, performance status or body composition in the preoperative period. 

**Figure 2:**
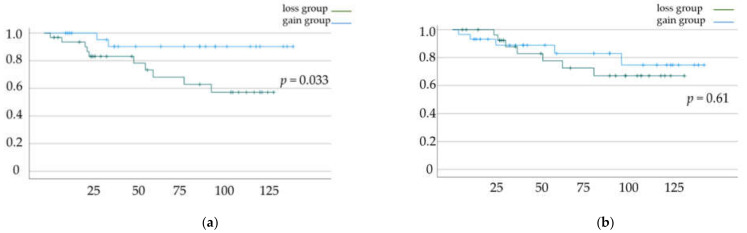
(**a**) Disease-free survival (DFS) and (**b**) overall survival (OS) rates according to muscle mass change. Kaplan–Meier curves and log-rank test.

**Figure 3:**
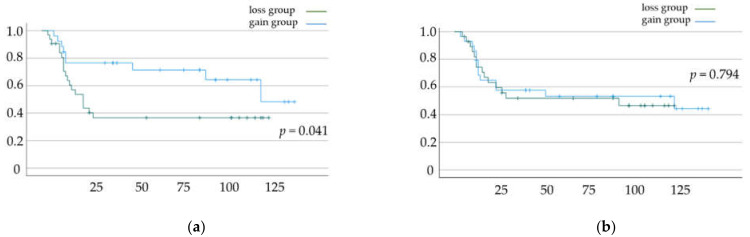
(**a**) Disease-free survival (DFS) and (**b**) overall survival (OS) rates according to total fat mass change. Kaplan–Meier curves and log-rank test.

DFS and OS rates based on the status of skeletal muscle mass change and total fat mass change are shown in Figure 2a,b and Figure 3a,b, respectively. Significant differences in DFS and OS were observed when patients were classified based on the status of skeletal muscle mass change (*p* = 0.033 (Figure 2a) and *p* = 0.041 (Figure 2b), respectively). In contrast, we did not find a significant difference in OS and DFS when patients were classified based on the status of total fat mass change (*p* = 0.61 (Figure 3a) and *p* = 0.794 (Figure 3b), respectively). 

## 4. Discussion

Coexisted sarcopenia reportedly affects cancer prognosis mainly in digestive and hepato-biliary-pancreatic (HBP) cancer [10,11,12,13,21,22,23]. In a previous report, we retrospectively evaluated the sarcopenic status during the preoperative period of patients who were Japanese and had ovarian cancer [14]. We found that, unlike other types of cancer, preoperative sarcopenic factors, such as decreased muscle volume and quality, were not associated with poor prognosis among patients with ovarian cancer in the Japanese population [14]. 

In the current study, we aimed to investigate whether changes in skeletal muscle volume occurred in patients with ovarian cancer during primary surgery and chemotherapy and whether these changes affect survival. We found that half of the patients experienced muscle volume loss and half of the patients experienced fat volume loss. Interestingly, changes in muscle mass and fat mass occurred independently. Among the clinical characteristics and pathological factors, only the presence of a residual tumor was associated with muscle loss. Association was not found between clinical stage and muscle loss when patients were divided into FIGO stage Ⅲ or less and stage Ⅳ. We also found that patients who gained skeletal muscle during treatment had a better prognosis than those who lost skeletal muscle. Conversely, no clinical characteristics or pathological factors were associated with loss of fat mass, and the change in fat mass had no effect on patient prognosis. 

However, the direct causality of this relationship remains unclear. During simple starvation, fat stores replace glucose as the primary fuel; consequently, a loss of fat mass generally precedes muscle loss [21]. In contrast, loss of skeletal muscle is one of the hallmarks of cancer cachexia [22]. In cachexia, metabolic and inflammatory changes are induced, and skeletal muscle wasting preferentially occurs regardless of fat mass wasting. Progression of cancer cachexia results in a worse prognosis [22]. Our findings are compatible with the reported progression of cachexia. In this study, the presence of residual tumor, a well-known prognostic factor, was related to muscle loss but not to fat loss in univariate analysis. This can be explained by the fact that muscle loss has been observed as a phenomenon of cachexia. Moreover, cancer treatments, such as surgery and chemotherapy, are invasive treatments for patients with cancer. Treatments may lead to reduced appetite, malnutrition, fatigue and subsequent inactivity. In addition, surgical in vivo stress may activate catabolism [15]. Cancer cachexia, together with treatment-related stressors, may contribute to specific body composition changes in patients with ovarian cancer. 

According to our previous findings, low skeletal muscle volume at a specific time point before surgery was not a prognostic factor [14]. 

In the current study, we measured skeletal muscle mass during the preoperative period and after the patients underwent primary surgery and chemotherapy. Interestingly, a comparison of patients who gained and lost skeletal muscle revealed that patients with a gain of skeletal muscle tended to have lower mean skeletal muscle mass at baseline (*p* = 0.070). Our results also showed that loss of fat mass and loss of muscle mass occurred independently. In addition, unlike skeletal muscle, loss of fat mass had no association with the patient’s prognosis, and preoperative fat mass had no association with subsequent changes in muscle mass. Some reports have shown an association between fat mass loss and shorter OS [19,23,24]. This discrepancy may be explained by the fact that two studies included different types of cancers, such as pancreatic cancer, and one study only reported ovarian cancer with advanced tumors. Our findings are concordant with those of previous reports on foregut cancer and lung cancer, in which patients who maintained or gained skeletal muscle had a longer OS than those who lost skeletal muscle. Additionally, baseline sarcopenia had no effect on survival [16,17]. One report on ovarian cancer in the European population showed results similar to our study, despite the difference in ethnicities [19]. As in our study design, the researchers included patients with ovarian cancer in all clinical stages and with all histological types and showed that skeletal muscle loss, but not preoperative muscle mass, has an effect on prognosis. Body compositional research, such as the current research, needs to consider ethnic differences; body composition has distinctive characteristics depending on ethnicity. In this regard, it is noteworthy that similar results were obtained between the current study and the study by Rutten et al. [19], regardless of ethnicity.

Compared with gastrointestinal (GI) and HBP cancers, the characteristics of ovarian cancer are distinctive in terms of susceptibility to cancer cachexia. The morbidity of cachexia widely depends on cancer types, with higher in cancer of digestive organs such as the GI, liver, and pancreas than other sites (40–80% vs. 0.5%) [25]. In addition, the prevalence of cachexia is generally lower in ovarian cancer than in cancers of the digestive organs. This may be attributed to the differing results regarding the relationship between preoperative skeletal muscle mass and prognosis. Unlike ovarian cancer, GI and HBP cancers, in which cachexia may exist in a relatively early clinical stage, may show a positive relationship between preoperative decreased skeletal muscle and poor prognosis. 

Our findings imply that measuring the change in skeletal muscle over time, rather than conducting sarcopenic evaluation only during the preoperative period, is important for patients with ovarian cancer. This may identify patients who need extra attention regarding their muscle mass and lead to an improved prognosis. Based on the univariate analysis in our study, the changes in the muscle mass of patients with residual tumors need to be closely followed. An important limitation of baseline measurement is that it is not associated with survival and cannot predict the future loss of muscle mass. In fact, some patients with low baseline SMI gained muscle mass during primary surgery and chemotherapy and were classified as having high baseline SMI after receiving treatment.

Some reports have shown that patients who have cancer and are obese tend to have a better prognosis even though obesity can be a contributing factor to some cancers, such as endometrial and colon cancer. This paradoxical phenomenon is called the “obesity paradox”, and its reliability is still controversial [26]. Our results did not show a beneficial effect of fat mass gained during cancer treatment on prognosis. Our previous report also showed no beneficial effects of preoperative fat mass on prognosis [14].

Our findings demonstrate the importance of muscle volume assessment throughout the disease course. The prevalence of cachexia rises along with the progression of the clinical stage in any type of cancer, regardless of the patient’s susceptibility to cachexia. Cancer cachexia progression is categorized into three stages according to severity: pre-cachexia, cachexia and refractory cachexia [22], which was associated with loss of muscle mass in a prospective study [27]. In this regard, muscle evaluation could help reduce skeletal muscle wasting before patients reach refractory cachexia, which is irreversible. 

Nutritional and rehabilitation interventions are the most important supportive care measures for patients with cancer to prevent the progression of cachexia [27]. Supportive care is effective in very advanced and refractory cachexia [27]. When patients lose muscle mass while undergoing treatment, nutritional support and rehabilitation interventions should be implemented to improve survival. Anorexia and reduced food intake are included in the definition of cachexia and may be caused by an invasive treatment process [22]. These would precede muscle loss. In addition to pharmaceutical intervention, nutritional counseling to promote not only a balanced diet and protein intake but specific nutrients such as eicosapentaenoic acid should be implemented [27]. Reportedly, interdisciplinary nutritional and exercise programs may improve the quality of life of cancer patients and should be incorporated into their standard care [28]. 

The putative effects of exercise on the prognosis of patients with cancer have been explored. The use of exercise can be a lifestyle modification in patients with cancer, with reported benefits of reprogramming the interactions between the host and the tumor microenvironment [29]. Briefly, understanding the role of myokine secretion from skeletal muscle may be the key to understanding the contribution of muscle loss to cancer-related mortality [30,31]. Some myokines, such as irisin and secreted protein acidic rich in cysteine (SPARC), are produced by exercise and are reported to have roles in suppressing tumor growth and improving mortality for several cancers [32,33,34]. These reports indicate that preventing muscle loss can activate myokines and improve cancer prognosis. 

The present study had a number of limitations. First, based on univariate analysis, the presence of a residual tumor was associated with loss of muscle mass. Due to statistical limitations, multivariate analysis for DFS and OS could not be performed using residual tumors as a confounding factor. There is a possibility that residual tumors might be a cause of muscle loss because a cancer cell mass causes chronic inflammation and results in muscle wasting as a process of cancer cachexia. Second, FIGO clinical stage has no association with the loss of muscle mass in univariate analysis. As mentioned previously, the prevalence of cancer cachexia generally increases along with the clinical stage. However, the definition of cancer cachexia does not include the clinical stage [22]. In this regard, it can be explained that loss of muscle mass is not necessarily associated with the clinical stage when muscle loss is a representative phenomenon of cancer cachexia. Third, we included patients with all clinical stages, from early to advanced stage.; this might have influenced the results. Fourth, the details of a surgical procedure, such as operation time and complications, were not evaluated though these factors may affect patients’ prognosis. Fifth, our study did not clarify whether the prevention of skeletal muscle loss can improve the prognosis of patients with ovarian cancer. To clarify the effect of providing support to address muscle loss, studies of therapeutic interventions for muscle wasting should be conducted prospectively. Sixth, patients were excluded if CT scans were not available, which may have resulted in a selection bias. Finally, this study was conducted retrospectively, in a single institution, with relatively small sample size. Therefore, these findings should be validated prospectively in a larger population. Multimodal interventions to stabilize or increase muscle mass and influence outcomes warrant further investigation.

## 5. Conclusions

Loss of skeletal muscle mass and fat mass occurs in approximately 50% of patients with ovarian cancer during primary debulking surgery and chemotherapy. In this study, these changes occurred independently of each other. Loss of skeletal muscle mass, but not fat mass, may be a prognostic factor in patients with ovarian cancer. It is important to closely monitor patients’ body composition, and nutritional and rehabilitative interventions should be provided to improve the prognosis of patients with ovarian cancer.

## Figures and Tables

**Table 1:** Baseline characteristics of patients.

Characteristics (*n* = 58)		Mean ± SD (%)
BMI		22.73 (3.82)
Age		60.82 (12.71)
Performance status		0.08 (0.38)
		**Median (IQR)**
Hospitalization period (days)		15.50 (10.25)
Number of days between scans (days)		225.00 (80.5)
		***n* (%)**
Histology	Serous	23 (39.7)
	Endometrial	14 (24.1)
	Mucinous	9 (15.5)
	Clear cell	12 (20.7)
FIGO stage	I	25 (43.1)
	II	7 (12.0)
	III	17 (29.3)
	IV	9 (15.6)

BMI—body mass index; FIGO—International Federation of Gynecology and Obstetrics; SD: standard deviation; IQR: interquartile range.

**Table 2:** The changes in muscle mass and fat mass after debulking surgery and chemotherapy.

	Baseline L3 Area in cm^2^ (Median ± SD)	Change in L3 Area in cm^2^ (Median ± SD)	Rate of Change in L3 Area in % 100 Days (Median ± SD)
Skeletal muscle area	82.38 ± 15.92	−4.72 ± 10.23 *	−2.55 ± 10.94 *
Total fat area	141.85 ± 95.04	−9.15 ± 48.23	−2.43 ± 25.35
Subcutaneous fat area	88.17 ± 51.42	−0.49 ± 33.81	−0.53 ± 29.44
Visceral fat area	58.76 ± 52.10	−8.50 ± 25.84	−8.10 ± 63.65

* Indicates significant changes in L3 area measurements between scans 1 and 2 (* *p* < 0.05 paired-samples *t*-test). L3—third lumbar vertebra.

**Table 3:** Prevalence of patients with loss/gain of muscle/fat volume during debulking surgery and chemotherapy.

Body Composition	Category	*n* (%)
Muscle mass volume	Loss	32 (55.2)
	Gain	26 (44.8)
Total fat mass volume	Loss	29 (50.0)
	Gain	29 (50.0)

**Table 4:** Prevalence of patients with loss/gain of muscle/fat volume in each FIGO stage.

FIGO Stage, (*n*)	Muscle Mass Volume		Total Fat Mass Volume	
	Gain, *n* (%)	Loss, *n* (%)	Gain, *n* (%)	Loss, *n* (%)
Ⅰ (25)	13 (52.0)	12 (48.0)	12 (48.0)	13 (52.8)
Ⅱ (7)	4 (57.1)	3 (42.9)	5 (71.4)	2 (28.6)
Ⅲ (17)	8 (47.0)	9 (53.0)	8 (47.0)	9 (53.0)
Ⅳ (9)	2 (22.2)	7 (77.8)	4 (44.4)	5 (55.6)

**Table 5:** Factors associated with muscle volume change.

	Muscle Gain (*n* = 26)	Muscle Loss (*n* = 32)	*p* Value
Age	60.03	61.46	0.674
BMI at diagnosis	22.63	22.82	0.848
Hospitalization period (days)	17.50	18.25	0.777
Performance status	0.00	0.15	0.123
Number of days between scans (days)	222.19	219.15	0.876
FIGO stage IV (%)	2/26 (7.6%)	7/32 (21.8%)	0.131
Residual tumor positive (%)	7/26 (26.9%)	18/32 (56.2%)	0.025 *
Skeletal muscle area (cm^2^) at diagnosis	78.76	85.12	0.132
Skeletal muscle index (cm^2^/m^2^) at diagnosis	33.39	36.48	0.070
Intramuscular adipose tissue content	−0.509	−0.513	0.918
Total fat area (cm^2^) at diagnosis	164.58	164.99	0.987
Subcutaneous fat area (cm^2^) at diagnosis	98.67	92.48	0.653
Visceral fat area (cm^2^) at diagnosis	65.90	72.50	0.636
Adipose tissue index (cm = /m^2^) at diagnosis	70.28	71.46	0.915

Student’s *t* and χ^2^ tests (* *p* < 0.05).

**Table 6:** Factors associated with total fat volume change.

	Fat Gain (*n* = 29)	Fat Loss (*n* = 29)	*p* Value
Age	58.93	62.72	0.260
BMI at diagnosis	22.78	22.69	0.935
Hospitalization period (days)	17.82	18.00	0.948
Performance status	0.00	0.17	0.091
Stage at diagnosis	1.93	2.41	0.113
Number of days between scans (days)	207.44	233.58	0.173
FIGO stage IV (%)	4/29 (7.7%)	5/29 (17.2%)	0.500
Residual tumor positive (%)	9/29 (31.0%)	16/29 (55.1%)	0.063
Skeletal muscle area (cm^2^) at diagnosis	84.24	80.30	0.351
Skeletal muscle index (cm^2^/m^2^) at diagnosis	35.60	34.59	0.556
Intramuscular adipose tissue content	−0.55	−0.46	0.076
Total fat area (cm^2^) at diagnosis	150.39	179.22	0.252
Subcutaneous fat area (cm^2^) at diagnosis	89.41	101.10	0.391
Visceral fat area (cm^2^) at diagnosis	60.98	78.11	0.214
Adipose tissue index (cm^2^/m^2^) at diagnosis	64.30	77.56	0.228

Student’s *t* and χ^2^ tests.

## Data Availability

Not applicable.
